# Aspartate Aminotransferase and Alanine Aminotransferase Detection on Paper-Based Analytical Devices with Inkjet Printer-Sprayed Reagents

**DOI:** 10.3390/mi7010009

**Published:** 2016-01-15

**Authors:** Hsiang-Li Wang, Chien-Hung Chu, Sing-Jyun Tsai, Ruey-Jen Yang

**Affiliations:** Department of Engineering Science, National Cheng Kung University, Tainan 70101, Taiwan; sunshinesun5815@yahoo.com.tw (H.-L.W.); as195735@yahoo.com.tw (C.-H.C.); andrey.tsai@gmail.com (S.-J.T.)

**Keywords:** paper-based analytical devices, biochemistry detection, low-cost, inkjet printer

## Abstract

General biochemistry detection on paper-based microanalytical devices (PADs) uses pipette titration. However, such an approach is extremely time-consuming for large-scale detection processes. Furthermore, while automated methods are available for increasing the efficiency of large-scale PAD production, the related equipment is very expensive. Accordingly, this study proposes a low-cost method for PAD manufacture, in which the reagent is applied using a modified inkjet printer. The optimal reaction times for the detection of aspartate aminotransferase (AST) and alanine aminotransferase (ALT) are shown to be 6 and 7 min, respectively, given AST and ALT concentrations in the range of 5.4 to 91.2 U/L (*R*^2^ = 0.9932) and 5.38 to 86.1 U/L (*R*^2^ = 0.9944). The experimental results obtained using the proposed PADs for the concentration detection of AST and ALT in real human blood serum samples are found to be in good agreement with those obtained using a traditional spectrophotometric detection method by National Cheng Kung University hospital.

## 1. Introduction

Liver disorders such as hepatitis and hepatocirrhosis are among the most common health problems globally, and are caused by many different factors, including excessive alcohol consumption, irregular sleep patterns, lack of exercise, irregular meal times, and an over consumption of high calorie foods [1,2]. In clinical testing, the levels of aspartate aminotransferase (AST) and alanine aminotransferase (ALT) provide important indicators of potential liver disease. In the blood serum of healthy adults, AST has a concentration of around 5 to 40 U/L, while ALT has a concentration ranging from 5 to 35 U/L [3]. However, when the liver, heart, or muscle is diseased or damaged, the levels of AST or ALT rise. Consequently, in detecting liver disease, effective methods for measuring the AST and ALT levels in human blood are urgently required. 

The concentrations of AST and ALT are generally evaluated using spectrophotometric [4,5], chemiluminescence [6], colorimetric [7,8], or chromatography [9] methods. However, such methods require the use of expensive bulky equipment and the intervention of skilled clinical staff. Accordingly, the problem of developing low-cost and simple paper-based analytical devices (PADs) for disease diagnosis purposes has attracted significant interest in recent years [10,11,12]. The first propose patterned paper as diagnostic platform by the Whitesides group. PADs comprise cellulose fiber as the main component, and hence sample transport is achieved by means of capillary forces alone without the need for external driving mechanisms such as pumps. PADs have many advantages compared to traditional bench top systems, including lower reagent costs, reduced sample volumes, faster detection time, lower cost, and greater portability. As a result, they have found increasing use in analytical and clinical chemistry, food safety testing, environmental monitoring, and others [13,14]. 

PADs can be produced through photolithography, handcrafting, cutting, or printing methods. Photolithography methods [15] require a UV lamp, hotplate, and a mask, and embed the photoresist into the paper by means of UV exposure through the mask. In handcrafted methods, the devices are fabricated via a manual wax drawing or stamping process [16]. Such methods are simple and require no expensive equipment. However, they have a poor resolution and low throughput. Cutting methods utilize computer-controlled knife-shaping [17] or laser cutting [18] techniques, and are thus ideally suited to mass production. However, the equipment cost is typically rather high. The other low-cost and repeatable cutting method to make PADs is craft punch patterning [19,20]. In wax-printing methods [21,22,23], wax is printed on the surface of the paper in the desired configuration and a heating process is then performed such that the wax melts and penetrates into the paper to form hydrophobic channels. Through a careful control of the wax quantity, the heating time, and the heating temperature, PADs with either hemi-channel or fully-enclosed channels can be easily formed [24]. Compared to photolithography, handcrafting, and cutting methods, wax printing is inexpensive, rapid, and extremely versatile. As a result, it is one of the most commonly used methods for producing PADs for both prototype and mass production purposes. 

In performing a biochemistry detection using PADs, the reagents and samples are generally applied using pipette titration. However, such an approach is extremely time-consuming for large-scale detection processes. To address this problem, several recent studies have demonstrated the use of inkjet printing technology in depositing biomaterials or functional materials in carefully defined regions of the paper surface [25,26,27]. Inkjet printing has many advantages for PAD manufacturing, including a rapid throughput, low cost, a straightforward process, good versatility, and the potential for mass production. Moreover, the cartridges of the printer can be filled with different reagents in order to realize multi-assay devices in a single printing run [28,29]. The present study uses a modified inkjet printer to spray biochemical reagents onto PADs for the detection of AST and ALT. The feasibility of the proposed approach is demonstrated by comparing the experimental results for the AST and ALT concentrations of human blood samples with the results obtained using a traditional spectrophotometric approach by National Cheng Kung University hospital.

## 2. Materials and Methods

### 2.1. Modified Inkjet Printer

The biochemical reagents were sprayed onto the PADs using an Epson T50 inkjet printer (Seiko Epson Corporation, Nagano-Ken, Japan). The printer was specifically chosen since it is a piezoelectric printer and thus, compared to thermal inkjet printers, avoids the risk of heat-induced damage to the reagent solution. In addition, the Epson T50 printer has a compact disc (CD) printing mode, and thus allows the printing region to be defined with a high degree of precision.

Circuit damage and nozzle chamber clogging may render the inkjet printer head inoperable. Thus, the exposed circuits in the printer head were sealed using Scotch tape (3M, St. Paul, MN, USA) to protect against accidental reagent leakage. In addition, clogging was resolved by injecting ethanol into the jet head until gas bubbles were no longer observed.

### 2.2. Fabrication of Paper-Based Analytical Devices

**Figure 1:**
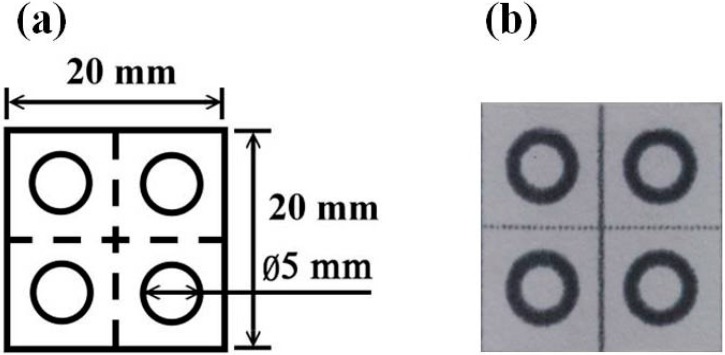
(**a**) Schematic illustration of paper-based analytical device; (**b**) Photograph of paper-based analytical device.

The PADs had dimensions of 20 mm × 20 mm and contained four circular regions; each with a diameter of 5 mm (Figure 1). (Note that the PADs were patterned with four detection regions in order to facilitate their mass production. However, biological detection was performed using PADs with one detection region only.) The PADs were designed using AutoCAD 2012 software (Autodesk, Marin, CA, USA) and printed on ADVANTEC chromatography filter paper (No. 51B, Advantec MFS, Inc., Tokyo, Japan) using a commercial wax printer (Fuji Xerox ColorQube 8570 solid ink printer, Fuji Xerox, Tokyo, Japan). Following the printing process, the PADs were placed in a furnace (Vulcan™ A-550, Dentsply, ON, Canada) and baked at 155 °C for 90 s to facilitate the penetration of wax into the filter paper. Finally, the reagent was sprayed onto the detection region of the PADs using the modified EPSON T50 printer described above. 

### 2.3. Preparation of Reagent Solutions

The AST reagent (Roche, Mannheim, Germany) comprised reagent 1 (R1) and reagent 2 (R2) solutions mixed in a ratio of 5:1. The R1 solution consisted of 100 mmol/L, pH 7.8 tris(hydroxymethyl)aminomethane (TRIS) buffer, 300 mmol/L l-aspartate, 0.23 mmol/L nicotinamide adenine dinucleotide (NADH), and 0.53 U/mL malate dehydrogenase (MDH). The R2 solution consisted of 75 mmol/L α-ketoglutarate. The equilibrium reaction between the reagent and the AST sample has the form shown below. The increase in oxaloacetate following the reaction process is determined via an indicator reaction catalyzed by malate dehydrogenase. More specifically, NADH is oxidized to nicotinamide adenine dinucleotide (NAD+). The reduction in NADH is directly proportional to the rate of formation of oxaloacetate and thus of the AST activity.

(1) $$\alpha \text{-ketoglutarate}+L\text{-aspartate}\overset{\text{AST}}{↔}L\text{-glutamate}+\text{oxaloacetate}$$

(2) $$\text{oxaloacetate}+\text{NADH}+H^{+}\overset{\text{MDH}}{↔}L\text{-malate}+{\text{NAD}}^{+}$$

The ALT reagent (Roche) also comprised R1 and R2 solutions mixed in a ratio of 5:1. The R1 solution consisted of 125 mmol/L, pH 7.3 TRIS buffer, 625 mmol/L l-alanine, 0.23 mmol/L NADH, and 1.5 U/mL lactate dehydrogenase (LDH). The R2 solution consisted of 94 mmol/L α-ketoglutarate. The addition of ALT enzyme prompts the equilibrium reaction shown below, in which the increase in pyruvate prompted by the ALT activity is determined in an indicator reaction catalyzed by lactate dehydrogenase.

(3) $$\alpha \text{-ketoglutarate}+L\text{-alanine}\overset{\text{AST}}{↔}L\text{-glutamate}+\text{pyruvate}$$

(4) $$\text{pyruvate}+\text{NADH}+H^{+}\overset{\text{LDH}}{↔}L\text{-lactate}+{\text{NAD}}^{+}$$

### 2.4. Reagent Spraying Process

**Figure 2:**
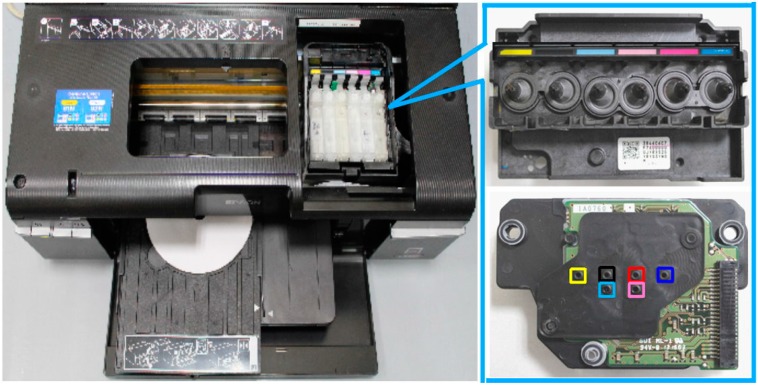
Epson T50 printer with six color channels.

**Figure 3:**
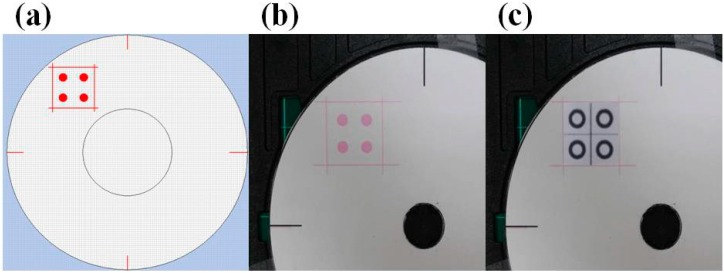
(**a**) Design of printing area using bespoke CD software; (**b**) Designated printing area on CD printing tray; (**c**) Analytical device attached to designated printing area.

As shown in Figure 2, the Epson T50 printer has six ink cartridges (*i.e*., six single color channels), namely yellow, black, light blue, light red, red, and blue. However, the RGB values of the light blue and light red color channels are not easily defined. Hence, these channels were not considered in the reagent spraying process. The print head contained 540 nozzles (each with an approximate diameter of 18.09 μm) arranged in the form of a 90 × 6 array. The printing region in the CD tray was defined using Epson CD Printing Software (Figure 3a,b). The PADs were then attached to the surface of a CD in the corresponding region and inserted into the printing tray (Figure 3c). Finally, the reagent was injected into the yellow ink cartridge in the printer head and sprayed on the papers.

### 2.5. Color Changes with Reaction Time

**Figure 4:**
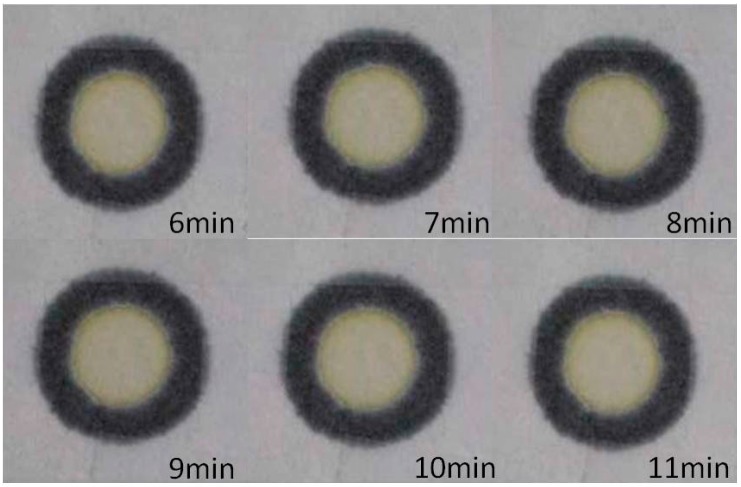
The color changes with reaction time for the AST reagent and human serum.

The color changes to be brighter with reaction time, a typical example is shown in Figure 4. In this case, we spray AST reagent onto the PADs with an inkjet printer and drip clinical human serum onto the reaction area of the PADs with a pipette. The images show the color changes with reaction time for the reagent and human serum from 6 to 11 min. The captured images were analyzed by the commercial software Adobe Photoshop 7.0 (Adobe Systems, San Jose, CA, USA). The Adobe Photoshop 7.0 software offers a built-in lasso function to highlight the reaction zone of the devices, for which the RGB color gradation was selected for analysis. In this case, we selected the R + G + B values as the representative color intensity, because the (Red + Green + Blue) R + G + B values showed better linear distribution with concentration. In the Adobe Photoshop 7.0 software, a darker color will yield a smaller intensity value. However, in order to show darker colors clearly, we used the value 200 to subtract the detection values to reverse the results. The formula is: reverse color intensity = 200 − color intensity. Therefore, the color intensity increased with concentration but decreased with reaction time. 

## 3. Results

### 3.1. Reagent Spraying Time

Table 1 shows the time required to spray 4, 16, and 86 devices, respectively. As shown, the time required to complete a 15-run spray process is 300, 600, and 1650 s, respectively. In other words, the average printing time per device (15-off) is equal to 75, 37.5, and 19.18 s, respectively. In other words, the feasibility of the inkjet reagent spraying method for mass production purposes is confirmed. 

**Table 1:** Inkjet printing time for 4, 16, and 86 devices.

Inkjet Printing Time	4 Devices	16 Devices	86 Devices
Time of print 15 runs (s)	300	600	1650
Average time of print a device (s)	75	37.5	19.18

### 3.2. Detection Results for PADs Prepared via Pipette Titration and Inkjet Spraying

**Figure 5:**
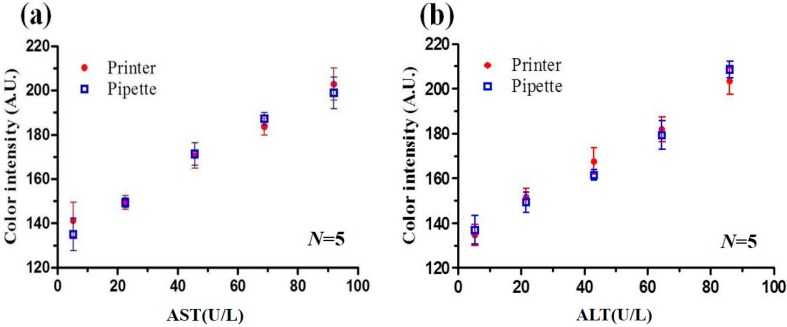
(**a**) Comparison of AST detection results obtained using printer and pipette reagent application methods; (**b**) Comparison of ALT detection results obtained using printer and pipette reagent application methods.

The feasibility of the inkjet reagent spraying process was investigated by comparing the detection results obtained using the sprayed PADs for AST and ALT samples with concentrations ranging from 5.4 to 91.2 U/L and 5.38 to 86.1 U/L, respectively, with those obtained using PADs prepared with a conventional pipette titration method. The corresponding results are shown in Figure 5a,b, respectively. Note that for each sample, the concentration is evaluated in terms of the mean R + G + B color intensity in the detection region of the PADs. It is seen that a good qualitative agreement exists between the two sets of results for each sample. Consequently, the basic feasibility of the proposed inkjet reagent spraying process is confirmed. 

### 3.3. Optimal Reaction Time

**Figure 6:**
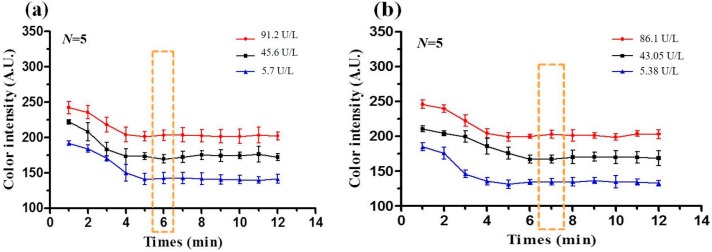
(**a**) Change in mean R+G+B intensity value over time for three AST concentrations (5.7 U/L, 45.6 U/L and 91.2 U/L); (**b**) Change in mean R + G + B intensity value over time for three ALT concentrations (5.38 U/L, 43.05 U/L, and 86.1 U/L). Note that dotted lines indicate the optimal reaction time in each case.

When the sample (AST or ALT) is placed on the detection region of the PADs, the ensuing reaction prompts a change in the color intensity over time. For practical detection applications, the optimal reaction time, *i.e*., the time at which the color intensity varies linearly with the sample concentration must first be determined. The variation in the color intensity was thus observed over time for AST and ALT samples with three different concentrations using a digital camera (Canon 650D, Canon, Tokyo, Japan). The captured images were processed using Adobe Photoshop 7.0 software to determine the corresponding change in the mean R + G + B intensity value in each case. Figure 6a,b shows the corresponding results for AST solutions with concentrations of 5.7, 45.6 and 91.2 U/L, respectively, and ALT solutions with concentrations of 5.38, 43.05, and 86.1 U/L, respectively. As shown, a linear relation between the color intensity and the sample concentration is obtained after approximately 6 min for the AST samples and 7 min for the ALT samples. In other words, the optimal reaction times for AST and ALT detection are equal to 6 and 7 min, respectively. 

### 3.4. Calibration Curves

**Figure 7:**
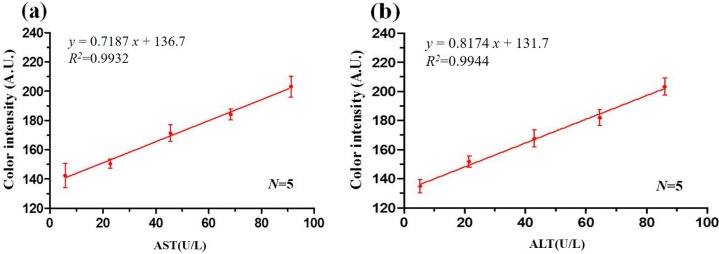
(**a**) AST calibration curve; (**b**) ALT calibration curve.

Figure 7a shows the measured values of the mean R + G + B color intensity after 6 min given AST concentrations of 5.7, 22.8, 45.6, 68.4, and 91.2 U/L, respectively. Similarly, Figure 7b shows the mean intensity values obtained after 7 min for ALT concentrations of 5.38, 21.53, 43.05, 64.58, and 86.1 U/L, respectively. (Note that prior to the detection experiments, the reagents and samples were placed in an ice bucket to prevent temperature-induced deterioration. For each sample, concentration detection was then performed under ambient temperature conditions (37 °C)). The correlation coefficient values of the AST and ALT detection results were found to be *R*^2^ = 0.9932 and *R*^2^ = 0.9944, respectively. Thus, the optimality of the AST and ALT detection times (6 and 7 min, respectively) is confirmed. In the blood serum of normal healthy adults, the AST concentration is around 5~40 U/L while the ALT concentration is around 5~35 U/L. Consequently, for the PADs developed in the present study, mean R + G + B color intensities greater than 166 (6 min) and 160 (7 min) represent possible indicators of liver disorder. 

### 3.5. Detection Results Obtained using Paper-Based Analytical Devices and Traditional Spectrophotometric Method

The feasibility of the reagent-sprayed PADs was further evaluated by comparing the AST and ALT detection results obtained for real human blood serum samples with those obtained using a traditional spectrophotometric method. (Note that the spectrophotometric assays were conducted by a certified test laboratory in National Cheng Kung University Hospital, Tainan, Taiwan.) Table 2 presents a qualitative comparison of the two methods in terms of the reagent/sample volumes and detection time. As shown, the spectrophotometric assay requires 180 μL R1 and 36 μL R2 reagents to detect each sample. By contrast, the PAD-based colorimetric assay requires a total reagent volume of just 2 μL. In addition, the colorimetric assay requires a sample volume of 2 μL (*i.e.*, the same as that of the reagent), whereas the spectrophotometric method requires a sample volume of 7 μL. In other words, the PAD-based colorimetric method reduces the reagent and sample volumes by 99.07% and 71.4%, respectively. 

**Figure 8:**
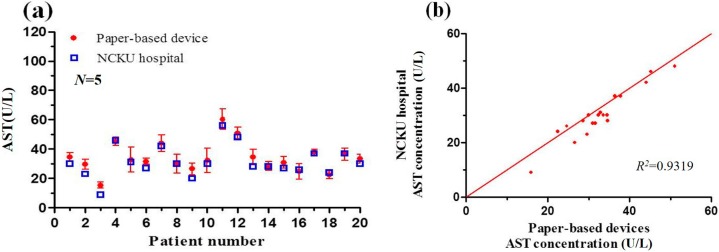
(**a**) AST concentration detection results obtained using paper-based analytical device and traditional spectrophotometric method from NCKU hospital; (**b**) Correlation between spectrophotometric results for AST concentration and paper-based analytical device results.

**Figure 9:**
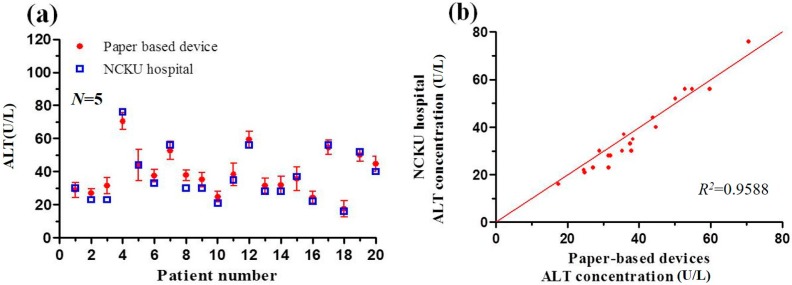
(**a**) ALT concentration detection results obtained using paper-based analytical device and traditional spectrophotometric method from NCKU hospital; (**b**) Correlation between spectrophotometric results for ALT concentration and paper-based analytical device results.

Figure 8 and Figure 9 compare the detection results obtained using the two methods for the AST and ALT concentrations of 20 human blood samples, respectively. It is seen that the correlation coefficient between the results obtained using the two methods has a value of *R*^2^ = 0.9319 for the AST sample and *R*^2^ = 0.9588 for the ALT sample. In other words, the practical feasibility of the proposed sprayed-reagent PADs is confirmed. However, it is observed that the detection results obtained using the PAD-based colorimetric method are generally higher than those obtained using the spectrophotometer. The light intensity of the shooting environment was found to be a very influential factor, and affected the picture colors. Hence, a fixed light source environment was needed to avoid any distortion in the image analysis results. 

**Table 2:** Comparison between colorimetric method and spectrophotometric method for detection of AST and ALT.

Methods	Roche	Computing and Microfluidics Lab
Detection method	Spectrophotometer	Paper-based devices
Volume of reagent	R1: 180 μL R2: 36 μL	R1 + R2: 2 μL (R1:R2 = 5:1)
Volume of sample	7 μL	2 μL
AST reaction time	10 min	6 min
ALT reaction time	10 min	7 min

## 4. Conclusions

This study has presented the use of a modified inkjet printer to spray biochemical reagent solutions onto paper-based analytical devices (PADs) fabricated using a wax-printing method. The feasibility of the proposed approach has been demonstrated by performing the colorimetric concentration detection of AST and ALT. The experimental results have shown that the optimal reaction time for AST detection is 6 min for AST concentrations in the range of 5.4 to 91.2 U/L (*R*^2^ = 0.9932). Similarly, the optimal reaction time for ALT detection is 7 min for ALT concentrations in the range of 5.38 to 86.1 U/L (*R*^2^ = 0.9944). In addition, it has been shown that a good agreement exists between the concentration detection results obtained using the sprayed-reagent PADs for real human blood samples and those obtained using a traditional spectrophotometer (*i.e.*, correlation coefficients of *R*^2^ = 0.9319 for AST and *R*^2^ = 0.9588 for ALT). However, the concentration results obtained using the colorimetric approach are slightly higher than those obtained from the spectrophotometric assay due to variations in the lighting conditions during the detection process. Consequently, it is recommended that the PAD calibration and assay processes should be performed using a fixed light source within a closed detection box. In addition, it is noted that the reagents and samples should be stored in a chilled environment prior to testing in order to prevent thermal deterioration under long-term room temperature conditions. 

In general, the results presented in this study confirm that the proposed inkjet reagent spraying method provides a low-cost, versatile, and effective method for the mass-production of paper-based analytical devices, and is thus ideally suited to the realization of point-of-care (POC) applications. 

## Supplementary Materials

The following are available online at http://www.mdpi.com/2072-666X/7/1/9/S1. Figure S1: Adobe Photoshop 7.0 software analyzed RGB values as color intensity. Figure S2: (a) Change in mean R + G + B values color intensity over time for AST; (b) Use the value 200 to subtract the detection values from (a) to reverse the results. Figure S3: (a) The result of AST detection obtained by printer method; (b) The result of AST detection obtained by pipette method. Figure S4: (a) The result of ALT detection obtained by printer method; (b) The result of ALT detection obtained by pipette method. Table S1: AST data. Table S2: ALT data.
