# Supplementary material for: Aspartate Aminotransferase and Alanine Aminotransferase Detection on Paper-Based Analytical Devices with Inkjet Printer-Sprayed Reagents
Source: Micromachines (Basel). 2016 Jan 15;7(1):9. doi: 10.3390/mi7010009 (PMC6189706; doi:10.3390/mi7010009)
Supplement: Supplementary file 1 [file micromachines-07-00009-s001.pdf]

Supplementary Materials: Aspartate Aminotransferase and Alanine Aminotransferase Detection on Paper-Based Analytical Devices with Inkjet Printer-Sprayed Reagents

Hsiang-Li Wang, Chien-Hung Chu, Sing-Jyun Tsai and Ruey-Jen Yang

To illustrate how to obtain the correlation between real images and colorimetric data, we give examples to show how to get results for Figures 5, 7 and 8.

Figure S1 shows a typical example of color changes with reaction time for the reagent and human serum. In this case, we spray AST reagent onto the PADs with an inkjet printer and drip clinical human serum (corresponding to patient number 12 in the Figure 8 of the manuscript) onto the reaction area of the PADs with a pipette. The images show the color changes with reaction time from 6 min to 11 min. Although these changes are hardly visible to the naked eyes the changes of RGB values are very clear with the commercial software Adobe Photoshop 7.0, mean value of R = 154.13, G = 152.7 and B = 114.35, respectively. In the Adobe Photoshop 7.0 software, higher color intensity will yield smaller intensity value. However, in order to show darker colors clearly, we used the value 200 to subtract the detection values to reverse the results, see Figure S2. Each case repeats 5 experiments in measurements.

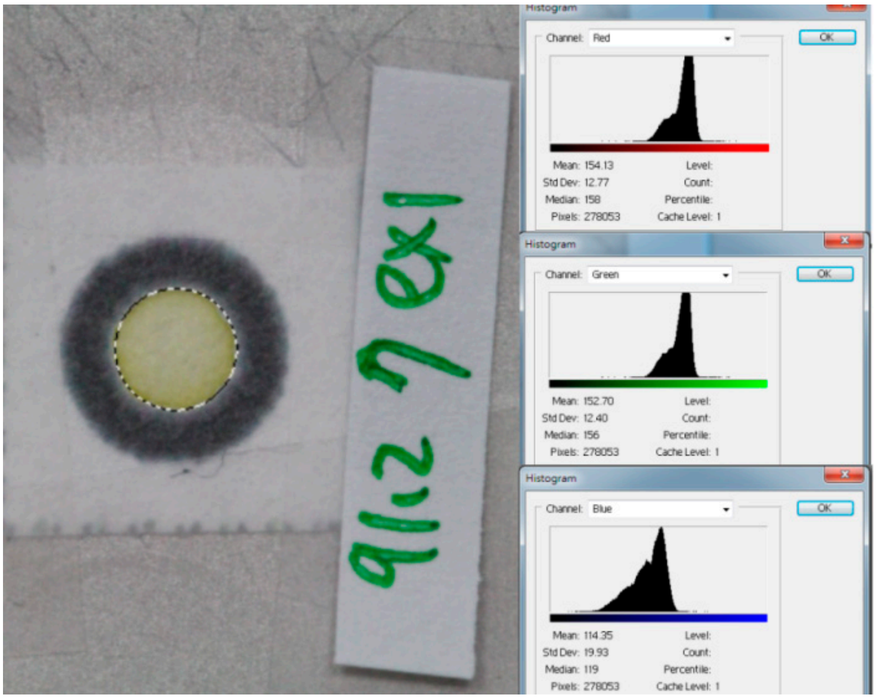

Figure S1. Adobe Photoshop 7.0 software analyzed RGB values as color intensity.

Similarly, an example is given below to show how to obtain results for Figure 5.

We give a typical set of images here. Figures S3 and S4 show the images of AST and ALT samples at concentration 44.05 U/L. Figure S3a is for AST detection obtained by the printer method (600-RGB = 168.58) and Figure S3b is that (600-RGB = 168.32) by the pipette method, respectively. Similarly, Figures S4a,b give the ALT value (600-RGB = 170.34) and (600-RGB = 166.98) by the printer and the pipette method, respectively. The RGB values are read directly from real images by the commercial software Adobe Photoshop 7.0. We can see the good qualitative agreement exists between the two methods. Although these changes in colors are hardly visible to the naked eyes but the results of the two methods are very clear with the commercial software Adobe Photoshop 7.0. Each case repeats 5 experiments in measurements.

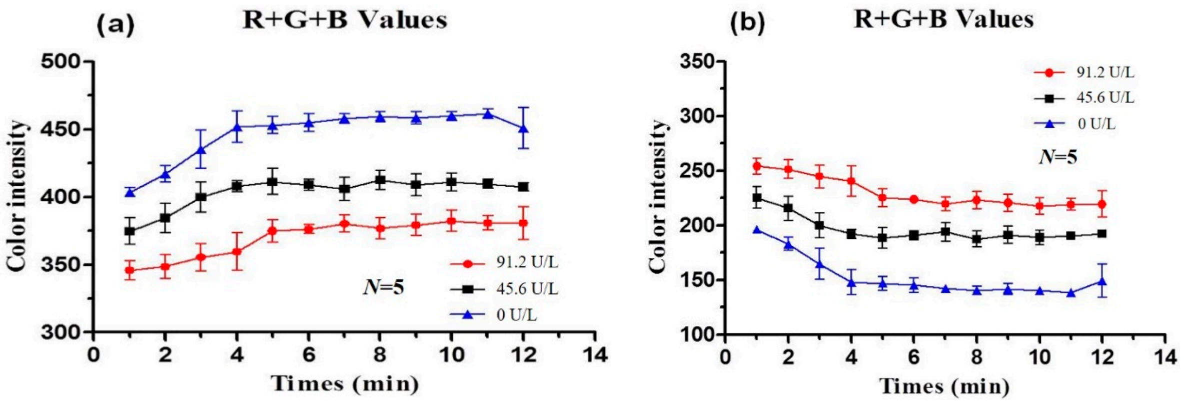

Figure S2. (**a**) Change in mean R + G + B values color intensity over time for AST; (**b**) Use the value 200 to subtract the detection values from (**a**) to reverse the results.

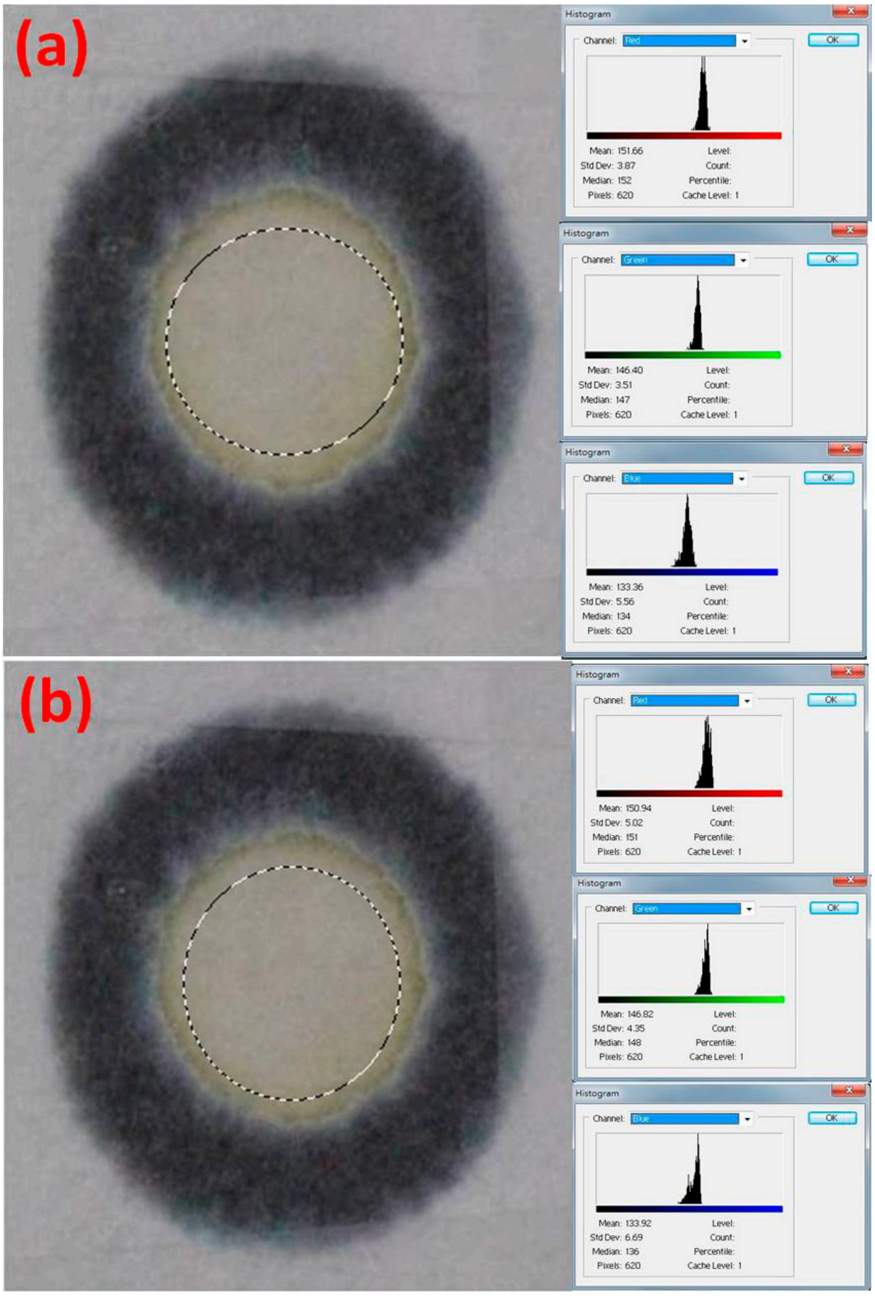

Figure S3. (**a**) The result of AST detection obtained by printer method; (**b**) The result of AST detection obtained by pipette method.

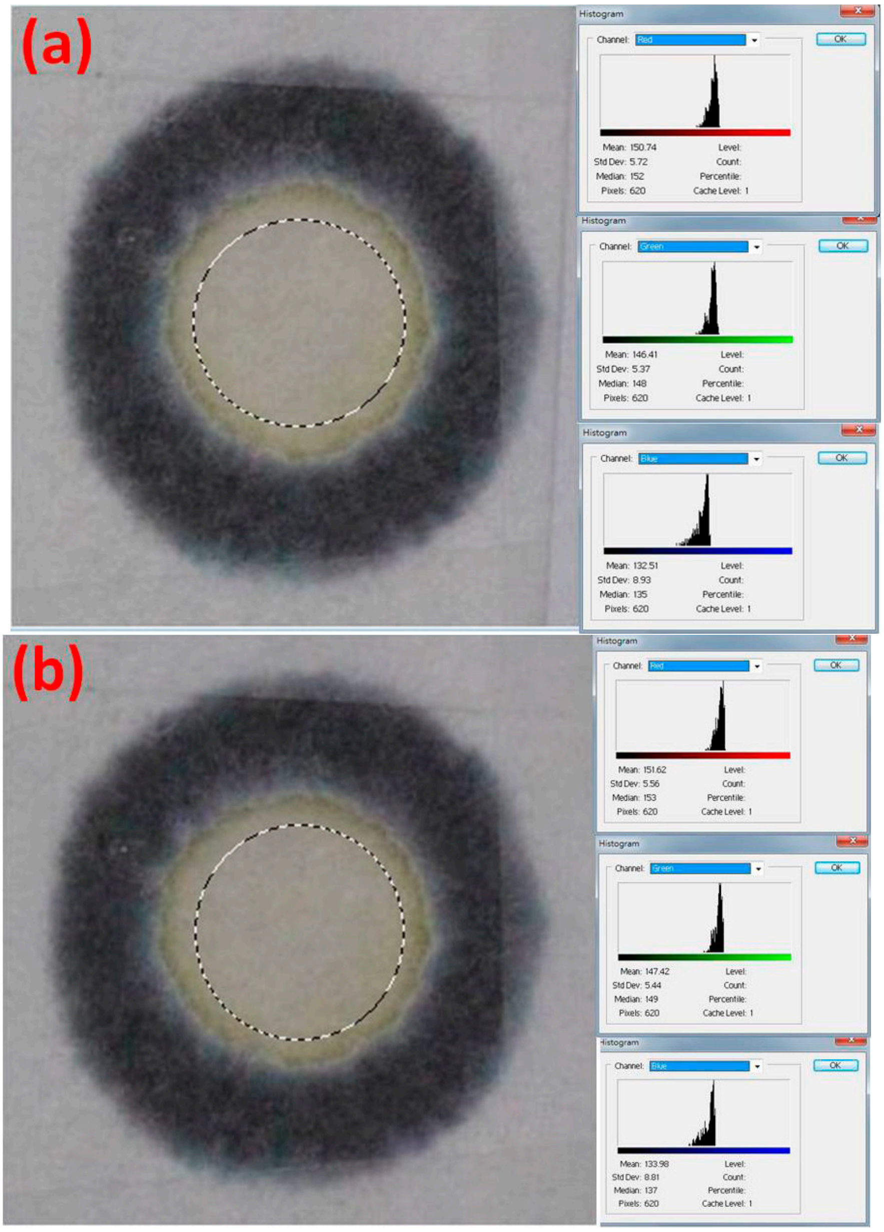

Figure S4. (**a**) The result of ALT detection obtained by printer method; (**b**) The result of ALT detection obtained by pipette method.

Tables S1 and S2 show the data for finding the linear regression relation for AST and ALT concentration in Figure 7, respectively. There are 5 repeats in experiment to measure data. AVE represents average of AST/ALT color intensity from the 5 measurements. SD is one standard deviation. The color on the paper-based device is composed of different proportions of R, G, B values. The R, G, B values change with the concentration and the reaction time. In this case, we selected the R + G + B values as the representative color intensity. And we obtain the calibration curves for different concentrations of AST and ALT by using the commercial software EXCEL.

Table S1. AST data.

AST (U/L)	$N = 1$	$N = 2$	$N = 3$	$N = 4$	$N = 5$	Color Intensity (R + G + B)	Standard Deviation
-	-	-	-	-	-	AVE	SD
5.7	148.73	139.38	145.67	148.73	130.15	142.53	7.91
22.8	149.06	150.29	152.16	148.95	156.46	151.38	3.11
45.6	180.22	169.48	166.84	172.56	170.81	171.97	5.05
68.4	186.47	188.25	187.46	183.25	179.58	185.01	3.57
91.2	193.53	198.45	209.81	204.15	209.88	203.16	7.16

AVE = Average of color intensity; SD (standard deviation) = $\sqrt{\frac{\sum(x-\bar{x})^2}{(n-1)}}$, ($n$= 5 measurements).

Table S2. ALT data.

AST (U/L)	$N = 1$	$N = 2$	$N = 3$	$N = 4$	$N = 5$	Color intensity (R + G + B)	Standard Deviation
-	-	-	-	-	-	AVE	SD
5.38	136.01	134.41	129.85	132.39	140.33	134.59	3.94
21.53	154.87	150.01	148.25	146.49	154.82	150.89	3.82
43.05	167.93	167.71	174.99	160.81	163.89	167.46	5.45
64.58	176.84	188.95	178.47	187.43	182.31	182.81	5.33
86.51	196.76	206.59	202.07	198.38	212.24	203.21	6.31

AVE = Average of color intensity; SD (standard deviation) = $\sqrt{\frac{\sum(x-\bar{x})^2}{(n-1)}}$, ($n$ = 5 measurements).
